# Strength Criteria for Cement-Treated Large-Size Macadam Base to Control Fatigue Failure

**DOI:** 10.3390/ma19132805

**Published:** 2026-07-01

**Authors:** Hongjiang Zhang, Di Wu, Xiangyu Li, Yao Yang, Jinpeng Du, Yingjun Jiang, Jinshun Xue

**Affiliations:** 1 Shaanxi Transportation Holding Municipal Road & Bridge Group Co., Ltd., Xi’an 710000, China; z1967422269@126.com (H.Z.); wd911127@163.com (D.W.); lxy1093677906@163.com (X.L.); xy1074256116@163.com (Y.Y.); jp765298643@163.com (J.D.); 2 School of Highway, Chang’an University, Xi’an 710064, China; 3 School of Civil Engineering and Architecture, Hubei University of Art and Science, Xiangyang 441053, China

**Keywords:** road engineering, CTB-50, fatigue failure, construction stage, operation stage, strength criteria

## Abstract

With a low cement dosage and a well-formed skeleton-dense structure, super-large-particle-size cement-stabilized macadam (CTB-50, maximum particle size of 53 mm) can effectively reduce base course cracking and construction costs. Nevertheless, the existing literature lacks research on the strength design criteria for CTB-50, and the absence of dedicated strength specifications currently limits its practical application. This study investigates the mechanical properties of CTB-50 and the stress levels in the (sub)base course under construction vehicle loading, based on the vertical vibration compaction method (VCM) and Miner’s fatigue cumulative theory. Aiming to prevent ultimate failure under a single load during construction and fatigue failure under repeated loading during both construction and operation, this study proposes strength criteria for CTB-50 to control fatigue damage. The 7-day compressive strength of CTB-50 specimens prepared using the VCM is approximately 90% of that of field core samples, whereas that obtained using the static-pressing method is less than 70% of the field core sample value. The mechanical strengths of CTB-50 specimens prepared via the VCM are highly correlated with those of on-site core samples. Based on the strength criteria for controlling ultimate failure during construction and fatigue failure during service, this paper proposes strength criteria for controlling the fatigue failure of CTB-50. Specifically, the 7-day splitting and compressive strengths of the base course for expressways and first-class highways should exceed 0.77 MPa and 7.6 MPa, respectively, while those of the sub-base course should exceed 0.71 MPa and 7.0 MPa, respectively.

## 1. Introduction

Currently, over 70% of pavement base materials used in China consist of cement-stabilized macadam CTB-30 (maximum particle size of 31.5 mm) [1]. Cracking is one of the major technical challenges associated with CTB-30 [2]. Existing studies have demonstrated that using larger particle sizes with lower cement contents in cement-stabilized crushed stone can provide higher mechanical strength, better crack resistance, and improved volume stability [3,4].

The meso-mechanical influence mechanism of cement-stabilized macadam with different nominal maximum aggregate sizes (NMASs) was investigated by Zhou et al., who showed that increasing NMAS delayed crack initiation, reduced the crack propagation rate, and decreased the total number of cracks at failure [3]. Reducing the cement dosage in cement-stabilized macadam can improve its overall crack resistance, although its strength and fatigue performance are slightly lower [4]. Furthermore, the rapid development of construction machinery technology has provided favorable conditions for the promotion and application of cement-stabilized macadam with super-large particle sizes [5].

Consequently, researchers have proposed the use of large-particle-size cement-stabilized macadam for pavement base courses. Increasing the coarse-aggregate particle size can enhance the skeleton interlocking force [3,6,7]; reducing the cement content can improve crack resistance [4]; and increasing the paving-layer thickness can reduce the number of layers, thereby enhancing the overall integrity of the cement-stabilized macadam base course [5]. These measures collectively aim to reduce cracking in cement-stabilized macadam and delay the development of pavement cracks. Jiang et al. conducted research on the mechanical properties of super-large-particle-size cement-stabilized macadam (CTB-50, maximum particle size of 53 mm), including compressive strength and splitting tensile strength [8,9]. They found that compared to traditional CTB-30, CTB-50 exhibited at least a 10% improvement in both compressive strength and splitting tensile strength. Moreover, under the same strength control criteria, CTB-50 required less cement, thereby reducing base course cracking and construction costs. These findings provided theoretical support for the engineering applications of CTB-50.

However, during the construction of CTB-50, when the curing time of the cement-stabilized macadam (sub)base course is insufficient and traffic is opened prematurely, the action of construction vehicles can readily cause early fatigue cracking of the cement-stabilized macadam material or even ultimate fracture under a single load [10]. Furthermore, repeated vehicle loads during construction can induce microdamage in the cement-stabilized macadam base course. This microdamage can accumulate and propagate under vehicle loads during the operational phase of the pavement, eventually leading to fatigue failure of the cement-stabilized macadam base course when a certain threshold is exceeded [11]. Therefore, to prevent both types of failure, CTB-50 must possess adequate load-bearing capacity and crack resistance, which necessitates appropriate strength design criteria for CTB-50 [12].

If the strength criteria for cement-stabilized macadam are set too high, a higher cement dosage will be inevitable for a given aggregate type, gradation, and compaction method to meet the strength requirements [13]. This will adversely affect the crack resistance and economic viability of the base course. Conversely, if the criteria are set too low, the bearing capacity of the base course will be directly compromised [14]. The current standard specifications are based on the heavy-compaction and static-compression methods [15]. However, studies have shown that the correlation between the engineering properties of specimens prepared using these methods and those of field core samples is, on average, less than 40% [16]. In contrast, the correlation between the mechanical strengths of specimens prepared by the vertical vibration compaction method (VCM) and those of field core samples can reach 93% [17]. Consequently, it is scientifically unsound to directly apply the existing standard specifications to the design and construction of VCM-compacted cement-stabilized macadam [18].

The existing literature lacks research on the strength design criteria for CTB-50. To address this gap, this study investigates the mechanical properties of CTB-50 and the stress levels in the (sub)base course under construction vehicle loading, based on the VCM and Miner’s fatigue cumulative theory. Aiming to prevent ultimate failure under a single load during construction and fatigue failure under repeated loading during both construction and operation, this study proposes strength criteria for CTB-50 to control fatigue damage. The research findings have significant practical implications for guiding the design and construction of CTB-50 base courses and for improving the crack resistance, bearing capacity, and economic efficiency of cement-stabilized macadam base courses.

## 2. Materials and Mixture Design

### 2.1. Aggregates

Five grades of aggregates—37.5–53 mm, 19–37.5 mm, 9.5–19 mm, 4.75–9.5 mm, and sand—were selected in this study. The technical specifications of the different aggregates are presented in Table 1 and Table 2.

### 2.2. Cement

The cement used in this study was P.O42.5 cement produced in Xi’an, Shaanxi. The technical specifications are presented in Table 3.

### 2.3. Aggregate Gradations

The gradation of CTB-50 used in this study is also presented in Table 4, and the cement content of CTB-50 is 3.0%.

## 3. Validation of Specimen Preparation and Testing Methods

**Figure 1:**
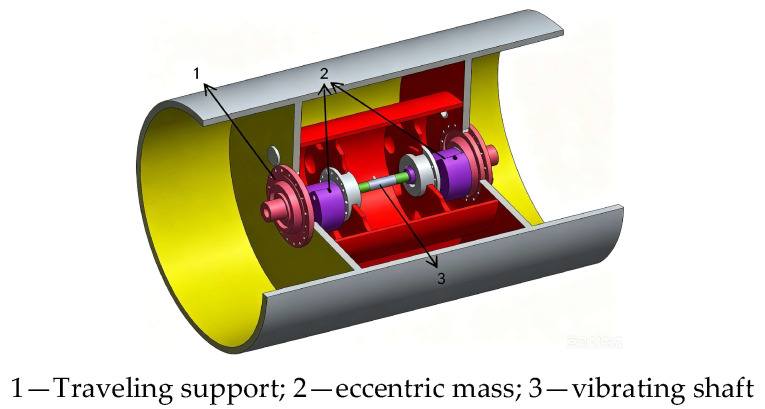
Schematic diagram of roller vibrator structure.

**Figure 2:**
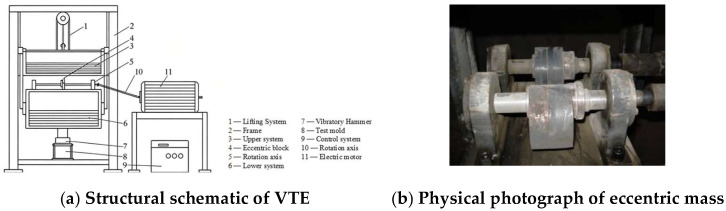
Schematic diagram of VTE structure.

The accuracy of simulating field compaction conditions using indoor specimen preparation methods directly affects the prediction accuracy of CTB-50 performance, which, in turn, influences the determination of its strength design criteria. The current standard specifications are based on the heavy-compaction and static-compression methods [15]. However, studies have shown that the correlation between the engineering properties of specimens prepared using these methods and those of field core samples is poor. Based on the working principle of the field vibratory roller (Figure 1) [19], our research group developed a vertical vibration compaction equipment (VTE) for CTB-50 specimens (Figure 2) and proposed the VCM for CTB-50 [20]. The operating parameters of this method are presented in Table 5.

In the VCM procedure, the optimum moisture content (OMC) and maximum dry density (MDD) of the mixture are first determined using the VTE. Subsequently, standard-sized specimens are prepared under the determined OMC and MDD conditions using the VTE. After curing to a specified age under standard conditions, the specimens are subjected to strength tests to evaluate their mechanical properties. In the conventional method, by contrast, the OMC and MDD of the mixture are determined using the heavy-compaction method. Standard-sized specimens are then prepared under the determined OMC and MDD conditions via static pressing on a static forming machine. After being cured under standard conditions to the specified age, the specimens are subjected to strength tests.

To evaluate the reliability of the VCM, a comparative analysis was conducted on the mechanical properties of CTB-50 specimens prepared using the conventional static-pressing method and the VCM. The results are presented in Table 6. Mechanical property tests were repeated six times, and the results were examined and averaged using Grubbs’ method.

As shown in Table 5, the mechanical strength of CTB-50 specimens prepared via the VCM is higher than that of specimens prepared by the conventional static-pressing method, and it also exhibits a stronger correlation with the mechanical strengths of field core samples. This difference is attributable to the distinct mechanisms of vertical vibration compaction and static pressing [21]. The vertical vibration compaction equipment was developed based on the field roller, making the preparation process of CTB-50 specimens more representative of field compaction conditions. During vertical vibration compaction, the vibrating hammer applies a sinusoidal excitation force to the compacted material, allowing the aggregates to undergo sufficient relative displacement [22]. This ensures adequate compaction without causing breakage of the coarse aggregates. The coarse aggregates form a skeletal structure that enhances the density and uniformity of the mixture, resulting in high specimen strength. In contrast, during specimen preparation by the static-pressing method, a vertical static pressure is applied, and the aggregates experience limited relative displacement during compaction. As a result, coarse aggregates—particularly those in the 37.5–53 mm range—are crushed, damaging the skeletal structure formed by their interlocking and compromising the specimen’s strength. Therefore, the mechanical strength of CTB-50 specimens prepared using the VCM is not only greater than that of specimens prepared by the conventional static-pressing method but also exhibits a higher correlation with field core strength. Consequently, the investigation of CTB-50 in this paper is based on the VCM.

The current strength criteria for CTB-30 in the specification are based on the heavy-compaction and static-pressing methods [18] and do not include criteria for CTB-50. Directly applying these existing strength design criteria to the design and construction of CTB-50 using the VCM is not scientifically justified. Therefore, based on the VCM and Miner’s fatigue cumulative theory and following investigations into the strength criteria for ultimate failure during construction and fatigue failure during operation, this study proposes strength criteria for controlling fatigue failure in CTB-50.

## 4. Mechanical Model and Construction-Stage Failure Strength Criterion of CTB-50

The CTB-50 base structure is assumed to consist of two layers: a sub-base and a base layer. After the CTB-50 sub-base is compacted and cured for a certain period, to achieve the required strength, the mixture-transport vehicles must pass over it during base construction. If the sub-base curing period is insufficient and the required strength is not achieved, the sub-base may suffer ultimate failure or fatigue damage under the load of construction vehicles. Therefore, the ultimate failure strength criteria during the construction period must be researched.

### 4.1. Mechanical Calculation Models and Parameters

**Figure 3:**
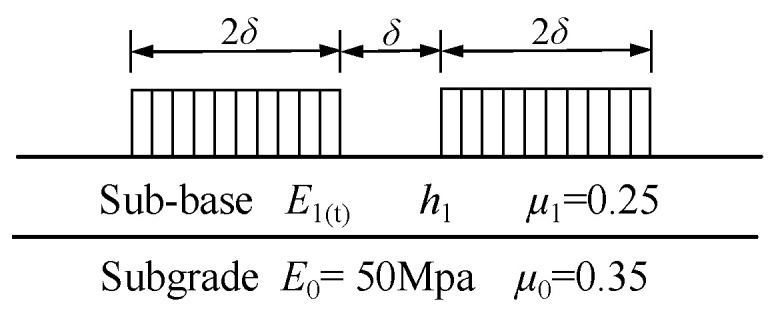
Simplified model for mechanical calculations.

In order to analyze the tensile stress and fatigue damage of the base layer under the action of construction vehicles, a simplified mechanical calculation model is presented in Figure 3, based on the assumption of complete interlayer continuity [23]. In the model, h1 represents the thickness of the CTB-50 substrate, δ is the tire radius, and μ is Poisson’s ratio.

### 4.2. Bottom Tensile Stress of the Sub-Base Layer Under the Action of Construction Vehicles

During the initial stage of CTB-50 formation, the initial strength is primarily contributed by physical effects and certain chemical interactions. As the curing age extends, cement hydration, setting, and hardening continue progressively, leading to the gradual formation of cement stone and an increase in the strength [24]. With ongoing hydration, the cement clinker is gradually consumed, slowing the rate of strength growth. Ultimately, when the cement clinker is nearly exhausted, the strength no longer increases but reaches its ultimate strength [25]. Previous studies have found that with increasing age, the growth curves of both the resilient modulus and splitting tensile strength of CTB-50 followed very similar patterns: the early strength growth rate was relatively rapid before 14 d, while after 90 days, the strength growth became very slow and tended toward a certain extreme value, i.e., the ultimate strength of CTB-50 [6,8].

The research group previously carried out a series of experimental studies to examine the influence of cement dosage (ranging from 1.5% to 4.0% at intervals of 0.5%) and curing duration (0, 3, 7, 14, 28, 60, 90, and 120 days) on the mechanical properties of super-large-particle-size cement-stabilized macadam CTB-50. Mechanical properties tests were repeated six times, and the results were examined and averaged through Grubbs’ method. Therefore, prediction models for the rebound modulus, splitting strength, and flexural tensile strength at different curing ages were constructed and calculated using Equations (1), (2), and (3), respectively [9].

(1) $$\frac{E_{\mathrm{cT}}}{E_{c\infty }}=0.28\;\left[\ln^{0.66}(T+1)+0.57\right]$$

In the formula, *E_cT_* and *E*_c∞_ represent the rebound modulus (MPa) and ultimate rebound modulus, respectively, of *T* (d) during the CTB-50 curing period, where *E*_c∞_ = 3800 MPa.

(2) $$\frac{R_{\mathrm{iT}}}{R_{i\infty }}=0.35\;\ln^{0.66}(T+1)\;$$

In the formula, *R_i_*_T_ and *R*_i∞_ represent the splitting strength (MPa) and ultimate splitting strength, respectively, of CTB-50 at the curing age *T* (d), where *R*_i∞_ = 1.55 MPa.

(3) $$R_{w}=1.4\;R_{i}$$

In the formula, *R_w_* and *R*_i_ represent the flexural tensile strength (MPa) and splitting strength (MPa), respectively, of CTB-50 at different curing ages.

Based on the above formulas, the calculated tensile stress results of the CTB-50 sub-base under the action of construction vehicles at different curing ages are presented in Table 7.

**Figure 4:**
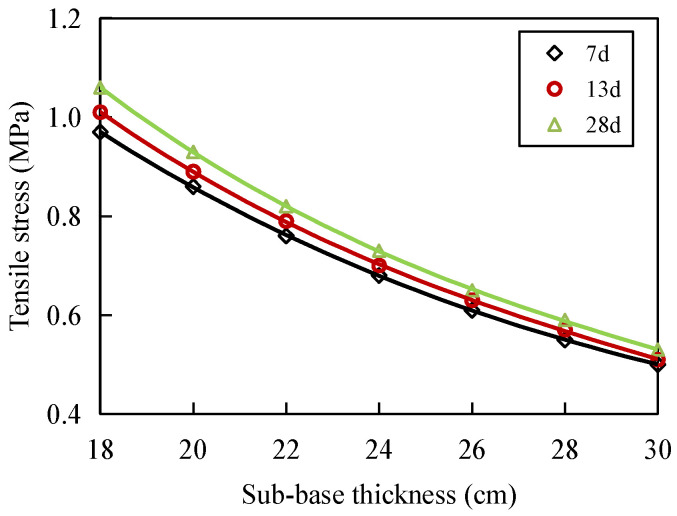
Tensile stress variation with sub-base thickness under different curing ages.

As shown in Table 7 and Figure 4, with increasing curing age, both the resilient modulus and flexural tensile strength of the CTB-50 sub-base increase; the tensile stress induced in the sub-base under vehicle loading also increases. Considering the requirements of construction progress, a shorter curing age for traffic opening is preferable; however, if the curing age is too short, the sub-base strength will be insufficient to resist the loads from construction vehicles, leading to cracking. Furthermore, as the thickness of the sub-base increases, the tensile stress at the bottom of the CTB-50 sub-base under construction vehicle loading will decrease significantly. Therefore, a greater thickness of the CTB-50 sub-base is advantageous. Nevertheless, a large layer thickness makes compaction of the CTB-50 mixture difficult. Based on comprehensive consideration of the mechanical properties of asphalt pavement CTB-50 sub-base and field construction feasibility, it is recommended that the design thickness of the CTB-50 sub-base be 18–26 cm, with a curing age exceeding 7 d.

### 4.3. CTB-50 Strength Criteria for Ultimate Failure During Construction

The flexural tensile strength and bottom tensile stress of CTB-50, presented in Table 6, reveal that when the design thickness of the CTB-50 sub-base is greater than 18 cm and curing age exceeds 7 d, the 7 d flexural tensile strength of the CTB-50 mixture should exceed 0.97 MPa to prevent ultimate failure of the sub-base under construction vehicle loading. The three-point bending beam test is traditionally used to measure the flexural tensile strength of cement-stabilized macadam mixtures; however, this test is complicated to perform and has stringent requirements regarding specimen preparation. Given that the VCM for preparing cylindrical specimens is simpler than that for beam specimens and that testing the splitting tensile strength and unconfined compressive strength of cylindrical specimens is easier than testing the flexural tensile strength and is more readily accepted in engineering practice, the 7 d flexural tensile strength criteria are converted into 7 d splitting tensile strength criteria and 7 d compressive strength criteria, according to Equations (3) and (4), respectively, as shown in Table 8. The 7 d splitting and compressive strength criteria in the table account for the fact that the actual strength of CTB-50 is approximately 0.92 times that of the laboratory-standard vibration-compacted specimen under standard curing conditions.

(4) $$R_{c\;7}=9.9\;R_{i\;7}\;$$

Here, *R_c_*_7_ and *R*_i7_ represent the compressive strength (MPa) and splitting tensile strength (MPa), respectively, of CTB-50 at a curing age of 7 d.

## 5. Fatigue Damage Accumulation Model and Operation-Stage Failure Strength Criterion of CTB-50

### 5.1. Miner’s Fatigue Cumulative Damage Theory and Basic Assumptions

In conventional fatigue tests, the cyclic stress remains constant throughout the test. However, most mechanical parts and structural components in service are subjected to spectrum-shaped working loads, meaning that the fatigue stresses they experience vary regularly. To mitigate the influence of differences in stress levels, Miner’s fatigue damage accumulation theory is adopted in engineering design.

Miner’s fatigue damage accumulation theory states that under a given stress level, if a component experiences complete damage or failure after *N* operating cycles, then under stress level *S*, after *n* cycles (where *n* is less than *N*), partial damage occurs. Assuming that each cycle causes identical damage, the damage ratio under *S* after *n* cycles will be *n*/*N*. If the component is subjected to different stress levels *Si*, a corresponding damage ratio *n_i_*/*N_i_* will be generated. When the sum of these damage ratios reaches 1, failure is predicted to occur, i.e., failure is expected when Equation (5) is satisfied:

(5) $$\frac{n_{1}}{N_{1}}+\frac{n_{2}}{N_{2}}+\ldots +\frac{n_{i-1}}{N_{i-1}}+\frac{n_{i}}{N_{i}}=1$$

Considering the complexity and extended duration of fatigue tests, this study only establishes the fatigue equation for CTB-50 at a curing age of 120 d, as shown in Equation (6). In the future, our group will complete systematic fatigue tests covering multiple maturation ages to verify the universality of this fatigue equation. It is assumed that the fatigue equations for CTB-50 at other ages conform to Equation (6):

(6) lg N=1.5717 − 21.0970 lg S

As the curing age of the CTB-50 base layer increases, its modulus and strength gradually increase. Consequently, under the same load, the stress level experienced by the CTB-50 base layer varies at different stages. In other words, the fatigue process of the CTB-50 base layer involves different stress levels. Therefore, it is assumed that the fatigue cumulative damage of CTB-50 follows Miner’s fatigue damage accumulation theory, i.e., Equation (5).

Investigation indicates that one stabilized soil-mixing plant typically supplies enough mixture for approximately 10 km of roadway and is usually located near the middle of that section, resulting in a one-way transportation distance of 5 km for delivery vehicles. Assuming the density of asphalt mixture is 2.51 t/m^3^ and that of CTB-50 mixture is 2.46 t/m^3^, trucks with a deadweight of 10 t and a carrying capacity of 20 t are used to transport the mixture [26]. Based on this, the cumulative number of BZZ-100 axle load applications from construction vehicles acting on each pavement structural layer can be calculated as shown in Table 9.

Based on above assumption, a 10 km section is considered a construction work unit. Assuming normal construction progress of approximately 600–700 m per day, the construction interval between two structural layers can be assumed to be 15 d.

### 5.2. Mechanical Calculation Models and Parameters

**Figure 5:**
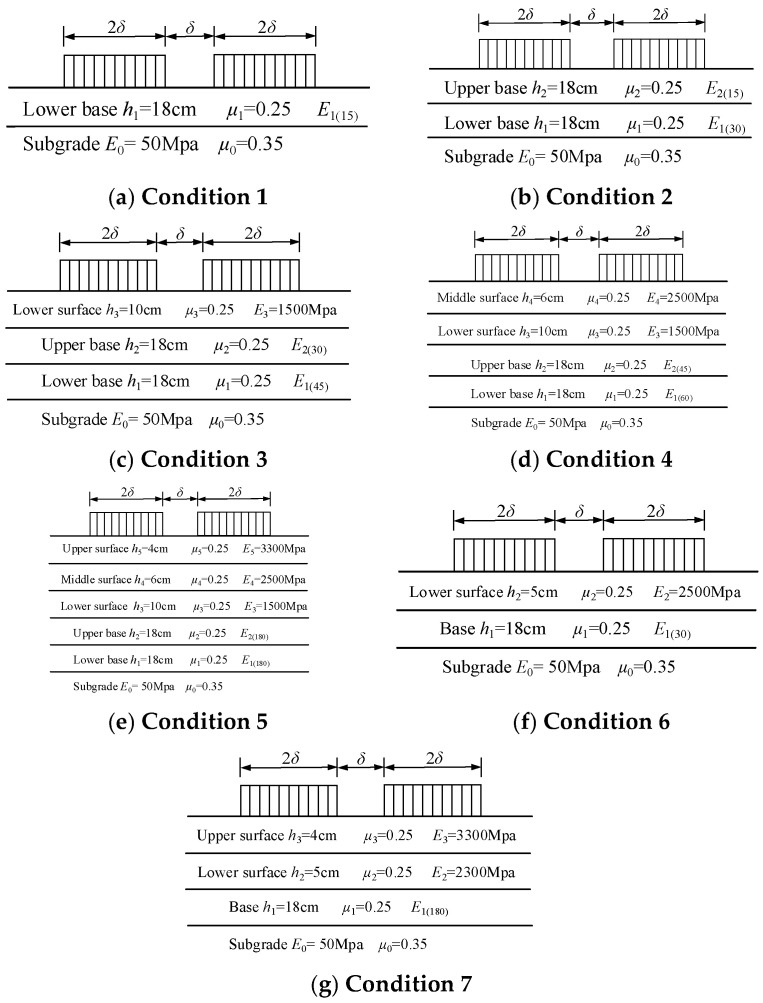
Mechanical calculation models at different stages.

The simplified mechanical calculation model for the CTB-50 (sub)base layer of the typical pavement structure at various stages is presented in Figure 5.

Working Conditions 1 to 5 in Figure 5 represent the mechanical calculation models for various stages of the CTB-50 base layer in typical pavement structures for expressways and first-class highways. Working Conditions 1, 6, and 7 represent the mechanical calculation models for various stages of the CTB-50 base layer in typical pavement structures for second-class highways. Each working condition is described as follows:

Working Condition 1: After the CTB-50 lower base layer is compacted and cured for a certain period (typically 7–10 d), construction vehicles for the CTB-50 upper base layer are allowed to travel on it. Working Condition 1 is the simplified mechanical model of a base layer under the action of construction vehicles for the upper base layer.

Working Condition 2: After the CTB-50 upper base layer is compacted and cured for a certain period (typically 7–10 d), construction vehicles for the asphalt pavement lower surface layer are allowed to travel on it. Working Condition 2 is the simplified mechanical model of the base layer under the action of construction vehicles for the lower surface layer.

Working Conditions 3 and 6: Within a certain period after the compaction of the lower surface layer, construction vehicles for the intermediate or upper surface layer are allowed to travel on it. Working Condition 3 is the simplified mechanical model of the base layer under the action of construction vehicles for the middle surface layer, and Working Condition 6 is the simplified mechanical model of the base layer under the action of construction vehicles for the upper surface layer.

Working Condition 4: After the middle surface layer of the CTB-50 base asphalt pavement is compacted and cured for a certain period (typically 7–10 d), construction vehicles for the upper surface layer are allowed to travel on it. Working Condition 4 is the simplified mechanical model of the base layer under the action of construction vehicles for the upper surface layer.

Working Conditions 5 and 7: These are the simplified mechanical model of the pavement under the action of transportation vehicles during the pavement operation stage.

The modulus of each structural layer of the asphalt pavement is presented in Figure 5. In the figure, x in Ex(t) refers to the structural-layer position, and t refers to the age of the CTB-50 base layer, calculated according to Equation (1), with the results presented in Table 10. Working Conditions 5 and 7 correspond to the pavement operation stage, where the modulus at the operation stage is taken as that at 180 days, with specific values taken from Table 10.

**Figure 6:**
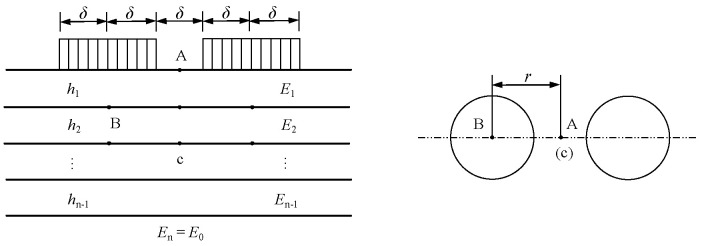
Diagram of road load and calculation points.

The calculation adopts the BZZ-100 load, with fully continuous interlayer bonding. The selection of calculation points is presented in Figure 6. The maximum tensile stress values at points B and C are taken as the tensile stress at the bottom of the base layer.

### 5.3. Load Stress of CTB-50 Base Layer Under Various Working Conditions

The calculated load stress results of the CTB-50 base layer under various working conditions are presented in Table 11.

As shown in Table 11, with increasing curing age and structural-layer thickness, the load stress on the CTB-50 base layer decreases sharply, while the flexural tensile strength of the cement-stabilized macadam increases continuously with age. This indicates that CTB-50 is more prone to damage during construction, and thus, overload control of construction vehicles should be strengthened during this period.

### 5.4. CTB-50 Strength Criteria for Fatigue Failure During Operation

The splitting strength is determined using Equation (2), and the stress level is subsequently defined as the ratio of the applied tensile stress to this flexural tensile strength. For a given stress level, Equation (5) yields the allowable number of load repetitions that CTB-50 can sustain before fatigue failure. The fatigue damage contributed by each axle load application is then computed as the ratio of the actual number of repetitions to the allowable repetitions, allowing the cumulative fatigue damage of the CTB-50 base layer to be assessed. Fatigue failure of the base layer is deemed to occur once the cumulative damage reaches unity. Accordingly, the splitting strength corresponding to a cumulative damage level within the range of 0.95 to 1.00 is recommended as the threshold (i.e., the ultimate splitting strength), below which the CTB-50 base layer remains free from fatigue-induced deterioration. Simulation results reveal that for expressways and first-class highways, when the 7 d splitting tensile strength exceeds 0.77 MPa, and for second-class highways, when the 7 d splitting tensile strength exceeds 0.69 MPa, the CTB-50 base layer will not experience fatigue damage within the designed service life. That is, the cumulative fatigue damage of the CTB-50 base layer reaches the range of [0.95, 1.00], as shown in Table 12.

Considering that the actual splitting tensile strength of CTB-50 is approximately 0.92 times the splitting strength of the laboratory-standard cured VCM specimens, the 7 d splitting strength criterion for CTB-50 can be obtained. This criterion is then converted into the 7 d compressive strength criterion, according to Equation (3), as shown in Table 13.

## 6. Proposed CTB-50 Strength Criteria for Controlling Fatigue Failure

Based on the above conclusions and actual conditions of engineering projects, the strength design criteria for CTB-50 to control fatigue cracking are proposed, as shown in Table 14. Table 15 lists the specified values for the 7 d unconfined compressive strength criteria for cement-stabilized material mix designs, according to JTG/T F20-2015 Technical Guidelines for Construction of Highway Pavement Bases [18].

A comparison of Table 14 and Table 15 reveals that the technical guidelines specify only the 7 d unconfined compressive strength criteria for cement-stabilized materials. In contrast, the strength design criteria for CTB-50 to control fatigue cracking, proposed in this paper, include two control indexes: 7 d splitting tensile strength and 7 d unconfined compressive strength. Moreover, the unconfined compressive strength criteria for CTB-50 are significantly higher than those in the guidelines. On the one hand, the nominal maximum aggregate size of CTB-50 is 53 mm, which is significantly larger than the maximum nominal aggregate size of 37.5 mm, stipulated in the guidelines. More coarse particles in the mixture result in greater stability of the compacted specimen and, thus, higher mechanical strength. On the other hand, the CTB-50 strength test specimens in our study were prepared using the VCM, while the guidelines require the static-compaction method. The mechanical strengths of the VCM specimens were approximately 1.5 times that of the static-compaction specimens, leading to higher strength criteria for CTB-50.

## 7. Field Validation

To verify the rationality of the proposed strength criterion for controlling fatigue failure of CTB-50, the CTB-50 base courses of Expressway A and First-Class Highway B were selected as research objects. Expressway A and First-Class Highway B are located in Zhengzhou and Xuchang, Henan Province, with design speeds of 100 km/h and 80 km/h, respectively. The CTB-50 strength criterion was adopted for the structural design of both base courses. Table 16 presents the 7-day unconfined compressive strength of CTB-50 specimens with varying cement contents.

**Figure 7:**
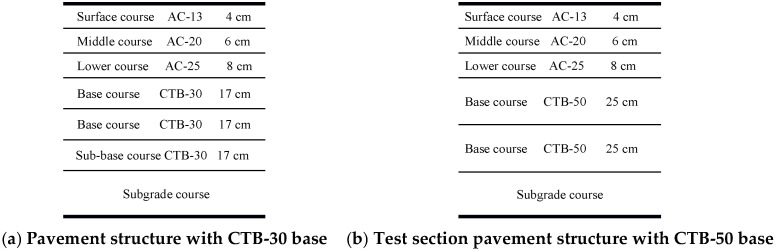
Pavement structure of Expressway A.

**Figure 8:**
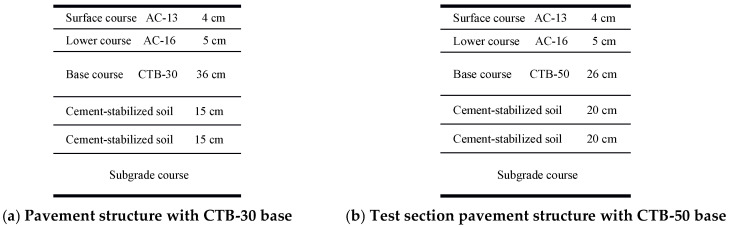
Pavement structure of First-Class Highway B.

**Figure 9:**
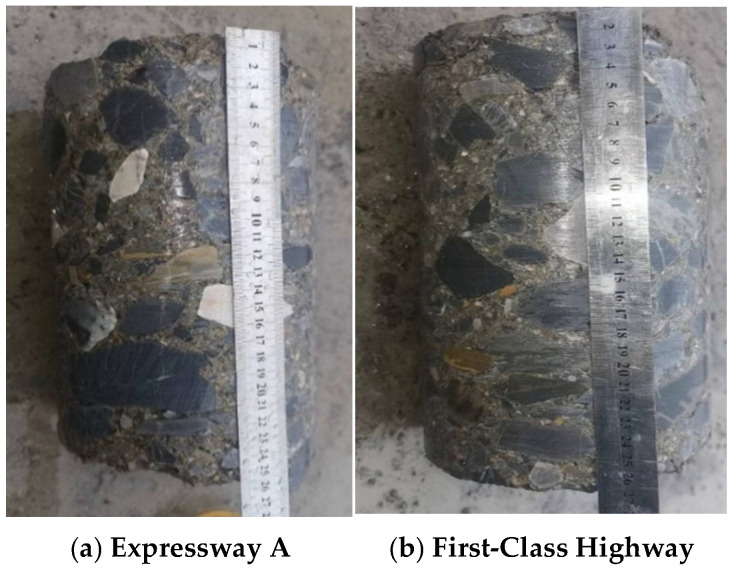
Core sample structures of different highways.

Table 16 indicates that the designed cement contents of CTB-50 base courses were determined as 3.3% for Expressway A and 3.0% for First-Class Highway B in accordance with the CTB-50 strength design criterion. Based on these design parameters, test sections with lengths of 500 m and 1200 m were constructed on the two roads, respectively. Schematic diagrams illustrating the pavement structures of the two test sections are presented in Figure 7 and Figure 8. The compactness of both test sections reached 99% during construction, and the core sample structures are displayed in Figure 9.

Conventional consensus holds that cracks within cement-stabilized macadam bases propagate to the asphalt pavement surface post-construction. Manual crack surveys were carried out two years after construction to characterize the anti-cracking capacity of CTB-50 base material. No cracks were detected within both CTB-50 test sections. In contrast, visible cracks of different severities appeared on the conventional CTB-30 cement-stabilized macadam sections, with crack spacing ranging from 20 m to 60 m. The field investigation results prove that the proposed strength design criterion for CTB-50 is reliable in practical engineering applications. Compared with the traditional CTB-30 cement-stabilized macadam base, the CTB-50 material can effectively mitigate cracking issues of pavement base courses.

## 8. Conclusions

This study develops novel two-stage strength criteria for CTB-50 materials to address both construction-stage ultimate structural failure and long-term operation-stage fatigue cracking risks, providing targeted design and quality control benchmarks for CTB-50 pavement base structures. The core research findings are summarized as follows:

(1) The vibrating compaction method (VCM) exhibits much higher reliability than the static-pressing compaction method for CTB-50 laboratory specimen preparation. The 7-day compressive strength of VCM-compacted specimens matches 90% of field core samples, while the value of static-pressing specimens is lower than 70%.

(2) The threshold strength indicators are determined to avoid construction-stage ultimate flexural failure induced by construction vehicle loads. The qualified 7-day strength limits of CTB-50 are 0.97 MPa for flexural tensile strength, 0.66 MPa for splitting tensile strength, and 6.5 MPa for compressive strength.

(3) Operation-stage fatigue failure criteria are quantified based on Miner’s fatigue cumulative damage theory to resist repeated traffic loads within the design service life. Higher strength thresholds are required for highways with heavier traffic loads: for expressways and first-class highways, the required 7-day splitting tensile strength and compressive strength are 0.77 MPa and 7.6 MPa; for second-class highways, the two corresponding strength thresholds are 0.69 MPa and 6.8 MPa. This graded design realizes refined fatigue resistance control matching actual traffic load conditions.

(4) Integrated fatigue cracking control strength criteria for CTB-50 are proposed by combining construction-stage and operation-stage failure control indicators. The proposed criteria show obvious differences from the existing standard specifications for conventional cement-stabilized pavement base materials. Compared with existing standard specifications for conventional cement-stabilized pavement materials, the proposed integrated two-stage strength criteria are more targeted for CTB-50 materials. The established criteria fill the blank of dedicated strength control standards for CTB-50 bases and can provide direct and quantitative guidance for material proportion design, construction quality inspection, and long-term service performance evaluation of CTB-50 pavement engineering.

## Figures and Tables

**Table 1:** The technical specifications of coarse aggregates.

Test Items	Test Values of Technical Specifications for Coarse Aggregates with Different Sizes (mm)				Required Values
	37.5–53	19–37.5	9.5–19	4.75–9.5	
Apparent relative density	2.786	2.761	2.731	2.729	≥2.6
Content of needle and flake particles (%)	2.1	7.1	12.9	--	≤15
Water absorption rate (%)	0.41	0.91	1.69	0.79	≤2.0

**Table 2:** The technical specifications of sands.

Test Items	Apparent Relative Density	Mud Content (%)	Crushing Index (%)	Methylene Blue Value (g/kg)
Test values	2.728	0.1	16	0.8
Required values	≥2.5	≤1.0	≤20	≤1.0

**Table 3:** The technical specifications of cement.

Test Items	Fineness (m^2^/kg)	Stability (mm)	3 d Compressive Strength (MPa)	3 d Flexural Strength (MPa)	Setting Time (min)	
					Initial Setting Time	Final Setting Time
Test values	336	1.3	22.8	5.4	212	452

**Table 4:** The gradation of CTB-50.

Sieve size (mm)	53	37.5	19	9.5	4.75	2.36	0.6	0.075
Percentage passing by mass (%)	100.0	70.0	60.0	42.0	34.0	26.0	14.0	4.5

**Table 5:** VTE working parameters of CTB-50 base layer mixture.

Working Frequency/Hz	On Board Mass/kg	Off Board Mass/kg	Static Eccentric Torque/kg·m	Nominal Amplitude/mm	Specimen Size/mm	Vibration Time/s
32	122	180	0.215	1.20	*Φ* 200 mm × *h* 200 mm	120

**Table 6:** Strength of CTB-50 specimens based on different compact methods.

Strength Items	Strength Test Values of Specimens Under The Following Methods (Mpa)			Specimens’ Strength Ratio of Different Methods	
	Core Samples	Static-Pressing Method Specimens	VCM Specimens	Static-Pressing/Core Samples	VCM/Core Samples
Compressive strength (MPa)	11.0	7.60	10.0	0.69	0.91
Splitting strength (MPa)	0.84	0.53	0.76	0.63	0.90

**Table 7:** Modulus, strength, and tensile stress of subgrade layer under vehicle load at different ages.

Items		Curing Age (d)							
		7	10	13	16	19	22	25	28
Modulus		2331	2502	2625	2722	2801	2869	2927	2978
Splitting strength		0.88	0.97	1.03	1.08	1.12	1.15	1.18	1.21
Flexural tensile strength (MPa)		1.23	1.36	1.44	1.51	1.57	1.61	1.65	1.69
Stress of different sub-base thicknesses (MPa)	18	0.97	1.00	1.01	1.02	1.03	1.04	1.05	1.06
	20	0.86	0.88	0.89	0.90	0.91	0.92	0.92	0.93
	22	0.76	0.78	0.79	0.80	0.80	0.81	0.82	0.82
	24	0.68	0.69	0.70	0.71	0.72	0.72	0.73	0.73
	26	0.61	0.62	0.63	0.64	0.64	0.65	0.65	0.65
	28	0.55	0.56	0.57	0.57	0.58	0.58	0.58	0.59
	30	0.50	0.51	0.51	0.52	0.52	0.53	0.53	0.53

**Table 8:** Strength standard of CTB-50 for controlling ultimate failure during construction.

Strength Index	7 d Flexural Strength (MPa)	7 d Splitting Tensile Strength (MPa)	7 d Compressive Strength (MPa)
Strength design criteria	≥0.97	≥0.66	≥6.5

**Table 9:** Required mixture and transportation vehicles for a single 5 km base layer.

Highway Class	Structural Layer	Thickness (cm)	Quantity of Mixture (t)	Number of Vehicles with Gross Vehicle Weight of 30 t (Vehicles)	Number of Axle Load Applications (Times)
Expressway, first-class highway	Upper sub-base	18	38,744.6	1937	5812
	Lower surface	10	13,805.0	690	2071
	Middle surface	6	8283.0	414	1242
	Upper surface	4	5522.0	276	828
Second-class highway	Lower surface	5	3921.8	196	588
	Upper surface	4	3137.3	157	471

**Table 10:** Modulus values of CTB-50 base.

Modulus Parameters	*E*_15d_	*E*_30d_	*E*_45d_	*E*_60d_	*E*_180d_
Values (MPa)	2692	3008	3187	3311	3800

**Table 11:** Load stress of CTB-50 base under various operating conditions.

Working Conditions	1	2	3	4	5	6	7
Sub-base bottom tensile stress (MPa)	1.024	0.412	0.304	0.245	0.222	0.582	0.684

**Table 12:** Fatigue damage of CTB-50.

Highway Class	Working Condition	Curing Age (d)	Tensile Stress (MPa)	Splitting Strength (MPa)	Flexural Tensile Strength (MPa)	Stress Level	Number of Cycles to Failure	Number of Axle Load Applications	Fatigue Damage	$\sum \frac{n_{i}}{N_{i}}$
Expressway, first-class highway	1	15	1.024	0.931	1.303	0.786	6057	5812	0.960	0.960
	2	30	0.412	1.072	1.501	0.274	2.6 × 10^13^	2071	7.83 × 10^−11^	
	3	45	0.304	1.152	1.613	0.189	7.1 × 10^16^	1242	1.76 × 10^−14^	
	4	60	0.245	1.207	1.690	0.145	1.9 × 10^19^	828	4.35 × 10^−17^	
	5	180	0.222	1.550	2.170	0.102	2.9 × 10^22^	2.5 × 10^7^	8.50 × 10^−16^	
Second-class highway	1	15	1.024	0.834	1.168	0.877	594	588	0.989	0.989
	6	30	0.582	0.960	1.345	0.433	1.8 × 10^9^	471	2.68 × 10^−7^	
	7	180	0.684	1.550	2.170	0.315	1.4 × 10^12^	2.5 × 10^7^	1.78 × 10^−5^	

**Table 13:** Strength criteria of CTB-50 based on fatigue failure.

Highway Class	Design Index	Design Criterion
Expressway, first-class highway	7-day compressive strength (MPa)	≥7.6
	7-day splitting strength (MPa)	≥0.77
Second-class highway	7-day compressive strength (MPa)	≥6.8
	7-day splitting strength (MPa)	≥0.69

**Table 14:** Strength design criteria of CT-50 based on fatigue failure.

Structural Layer	Highway Class	Compaction Degree (%)	7-Day Splitting Strength (MPa)	7-Day Compressive Strength (MPa)
Base	Expressway, first-class highway	≥98%	≥0.77	≥7.6
	Second-class highway	≥97%	≥0.71	≥7.0
Sub-base	Expressway, first-class highway	≥98%	≥0.69	≥6.8
	Second-class highway	≥97%	≥0.61	≥6.0

**Table 15:** Seven-day compressive strength design criteria of CTB-50 base on JTG/T F20.

Structural Layer	Highway Class	Extremely Heavy and Exceptionally Heavy Traffic	Heavy Traffic	Medium and Light Traffic
Base	Expressway, first-class highway	5.0~7.0	4.0~6.0	3.0~5.0
	Second-class highway	4.0~6.0	3.0~5.0	2.0~4.0
Sub-base	Expressway, first-class highway	3.0~5.0	2.5~4.5	2.0~4.0
	Second-class highway	2.5~4.5	2.0~4.0	1.0~3.0

**Table 16:** Seven-day compressive strength of CTB-50 with different cement content.

Highway Class	7-Day Compressive Strength (MPa) of CTB-50 with Different Cement Content (%)		
	2.0	3.0	4.0
Expressway A	5.9	7.2	8.6
First-Class Highway B	6.6	7.7	8.9

## Data Availability

The original contributions presented in this study are included in the article. Further inquiries can be directed to the corresponding authors.
